# Mr. Surr's Account of Horses Bitten by a Dog

**Published:** 1810-01

**Authors:** J. Surr

**Affiliations:** Clerkenwell


### Medical and Phyfical Journal.

VOL. XXIII.] JANUARY, 1810. [NO. 131.

*Printed for R. PHILLIPS, by W. Thorne, Red Lion Court, Fleet Street, London.*

### To Dr. ADAMS.

DEAR SIR,

IN compliance with your wishes, I have used all diligence in collecting facts concerning the horses that died, or were killed, after being bitten by a supposed rabid dog. You will, perhaps, think me tediously minute in some parts; but as all that is related has been collected with the most scrupulous exactness, it appeared that your record would be more valuable in proportion as every part should be supported by collateral evidence.

I am, &c.

J. SURR.

*Clerkenwell,* 13 *Dec.* 1809.

THE horses ascertained to have been bitten were five; whether the number was greater, is not certain, rumour speaking of one or more in the Borough. Those under consideration were all attacked between Shoreditch church and the bank of England. The dog which bit the whole was a sheep dog, of the long haired breed; as all were bitten in the nose or lips, the habits of the dog may perhaps account for the uniformity of his attack.

The first notice which the writer could obtain of him, was his biting a dog belonging to Mr. Harper, butcher, near Shoreditch church, on Saturday, October 21, about seven in the morning. From the dog he flew on a horse belonging to Mess. Risdon and Townsend, Curtain Road, standing within a few yards, in the shafts of a cart. He displayed much pertinacity in his design, making a third leap after two unsuccessful ones; by which he caught hold of the nose, and remained suspended nearly a minute. After detaching himself, he ran up one of the roads, seized and shook a bitch by the side of a cart. In a little while he returned by Old Street Road, thence to Shoreditch, ditch, and proceeded into Bishopsgate Street, where he seized a horse belonging to Mr. Ashenden, Cumberland Street, Curtain Road. By the Vine Inn he bit a horse belonging to Mr. Gray, the Royston carrier. In Wormwood Street he bit one belonging to Mr. Teanby, Old Street; displaying the same pertinacity in retaining his hold, the horse being compelled to strike him off with his fore feet. Lastly, he ran into Threadneedle Street, seized by the upper lip a mare in a cart belonging to Mr. Crow, butcher, and standing across the street. His furious manner excited the attention of the shopmen, who sallied out after him, towards the Bank, opposite which, by the aid of one of the labourers at the Auction Mart, he was killed. He was taken to Mr. Crow's yard, and remained there some time after the death of his mare; but before the writer could call for information, had been removed; a circumstance to be regretted, as the comparison of any morbid appearances with those in the horses, might havepointed to a similarity or dissimilarity as to the nature and seat of the disease. Mr. Harper caused his dog to be drowned as speedily as possible; but of the bitch no account has been obtained. It has not been ascertained that he attacked any human subject.

Let us now attend to all the circumstances in the order they occurred; first, with respect to Messrs. Risdon and Townsend's horse.

Mr. Harper the butcher being a spectator, and persuaded as to the madness of the dog, immediately sent the horse home; so that in about three quarters of an hour the bitten parts were freely cut out by Mr. Jones, veterinary surgeon in the Curtain Road; the wound healed without trouble, and to appearance soundly; a well closed seam only remaining, and comparatively without any cicatrizing hardness.

The horse was a black gelding about seven years old; of a disposition so gentle and docile, that he would follow the ordinary stable attendants, when bid to do so.

His owner, Mr. Townsend, aware of the circumstances, had kept his eye over him, and found that after a fortnight he fell off from his food, though he continued to eat more or less. He continued his work also till the evening of Saturday the twenty-first day after being bitten, but fell down suddenly whilst shooting the last load. This caused him to be attended to with care, and placed in a stall slightly separated from the other horses, but in the same stable.

On

On Sunday morning, Mr. Townsend found him very ill; offered him some water, which he seemed desirous of drinking; filled his mouth several times with it, but suffered it to escape; was chiefly intent on rubbing his nose against the pail and manger, displaying signs of uneasiness and impatience, such as pawing, restlessness, &c. He was from this day to the last, drenched in a profuse perspiration.

On Monday morning, about four o'clock, he was found on his back in the stall, much agitated, and pushing his legs to and fro with spasmodic vehemence. From this time till about two on Tuesday afternoon, there was no variation in symptoms, except that all progressively increased in violence.

On Tuesday, the twenty-fourth day from the bite, about twelve o'clock, he was seen by the writer. The symptoms, by account of the horse-keeper and others, were intense restlessness and agitation; that he had been secured in the stall by two halters, but during the forenoon had thrown himself backward, so as to induce the horse-keeper to liberate him lest he should strangle himself. Thus set at liberty, and having the range of a large stable to himself, he was perpetually desirous of changing his place or posture, and even galloped its whole length; he had, however, so far the command of himself, as to be kept in one end of the stable, by the sight of the cart-whip, except when exacerbations of the symptoms caused him to break all bounds. Such was the account given about Tuesday noon as above-mentioned; it was then not deemed prudent to go near him, the keeper having reported a disposition to run at those who placed themselves before him, or in his way; of this, however, I saw no appearance.—The stable was tolerably light.

The general restlessness and irritability were intense, causing him perpetually to change his situation, lying down, rising on his feet, rolling on his back from side to side; careless of his positions and actions in such a manner, as to cause an idea, that he must mean to hurt himself, and this in every change of posture. The perspiration had never ceased from Sunday preceding.

When on his feet, he was perpetually attempting to rub the hind part of the thighs against the manger, a symptom attending him from the beginning. When on the ground, his rollings appeared to have reference to the loins, his back being bent so as to throw his weight thereon; after each turn, the penis, though a castrated horse, was propelled out of the sheath, at least four or five inches, and ejected a few ounces, perhaps half a pint, of urine with all the vehemence of a squirt; this action, evidently a painful one, and causing great tension for the moment, was observable from the beginning.

Whether on his feet or reclined, he rubbed his nose incessantly against the manger, or amongst the litter, and in the latter case seemed desirous of retaining his nose immersed in it: this was a prominent symptom. After rubbing a while, jerking his head forward with great violence. It was said he had been observed to bite at the manger, but I was at that time out of the stable.

The eyes had a dull glazed appearance, and seemed so devoid of lustre, as to give an idea of his being blind, but that was not the case: he readily understood the motions of the whip.

He laboured under great difficulty of breathing, and frequent cough, both of which appeared to have occasional exacerbations. At these times, his actions broke all control, and became violently extravagant.

A frothy or semi-mucous saliva was observed about the mouth, from which it occasionally dropped without coughing: the cough commonly forced some away.—The breathing resembled the whizzing, without the sonorousness, of croup; the cough was facilitated in its passage by a particular attitude of the body and bend of the neck, so as to give an appearance as if he were desirous it should pass along the windpipe, without scouring it. All these symptoms increased greatly during the above-mentioned exacerbations.

When disposed to lie down, he threw himself at least half the way, as if from impatience, but apparently with difficulty from rigidity of the limbs, or from some internal sensation, or both.

During every exacerbation, the limbs were spasmodically affected; the stricture of the throat was more apparent, the breathing loud, or resembling that of a horse pinched on the windpipe to ascertain the soundness of his lungs. A pail of water being put before him in this state, he presently approached it, put his nose into it, and filled his mouth, without, however, swallowing any; then put his foot into it, and threw it over. There was irritation enough in his appearance to lead one to expect intense thirst. The symptom then, which has been held as of almost exclusive importance in Hydrophobia, was not prominent here, though it obtained in a degree from the beginning.

Though

Though disposed to actions so extravagant, they appeared rather to resemble such as might arise from intolerable suffering, than the result of madness guided by the will. But his threatening appearance to those who stood before him, and his subjection to the cart-whip, had less the appearance of delirium. His actions on the whole, however, might easily lead an ordinary bystander to give his disease the name of madness, i. e. of furious derangement.

The urgency of the symptoms, the increasing ungovernableness of his actions, the necessity of having the stable cleaned before evening, and the conjectured impossibility of recovery, induced his owner to have him shot, about two in the afternoon, on Tuesday, the twenty-fourth day after he was bitten.

The carcase was examined about twenty-four hours after death, in the presence of Dr. Adams, Mr. Taunton, two of Mr. Taunton's pupils, Mr. Jones, and Mr. Charles Newport, veterinary surgeons, and the writer. Though we had the opinions of the two last named gentlemen, the diseased parts were compared with similar ones taken from two or more other horses, one of which was killed, the other had died of some disease. The opinions of the horse-slaughterers were also attended to, where they could be of use.

The following were the appearances.

The abdomen had become distended with air; the skin still shewing marks of the profuse perspiration.

The blood had hardly coagulated, excepting in the heart, where a firm coagulum was found. It was remarkably black and thin, fluid even to the smaller vessels; and little having been drawn off when he was shot under great muscular exertion, every part exhibited marks of high injection. When exposed to the air it speedily reddened.

Inflammation was found in the mucous membranes of the stomach, windpipe, œsophagus or weasand, bladder, urethra, and back part of the mouth.

No inflammation was discovered in any serous membrane; all were exposed.

The brain healthy, veins on its surface injected; the dura mater of a natural appearance except slightly injected, without inflammation.

The membrane lining the upper part of the mouth, nearest the larynx, highly injected, though but slightly inflamed; and that inflammation seated in the finer capillary vessels prominent on its surface. This inflammation was continued, in the same degree, down the œsophagus to its entrance into the stomach, and down the windpipe, to within about six or more inches of its ramifications into the lungs; the œsophagus the least inflamed.

The greater part of the inner surface of the stomach affected with inflammation in various stages of forwardness: from the simple injected, to gangrene—of various colour, from blush red to livid and black, and in one or two points exhibiting incipient sphacelation; in the worst case not affecting the subjacent or muscular coat; no coating of coagulable lymph; the outer surface, as well as that of all the intestines, shewing no marks of disease, except six or seven dark coloured spots in their whole length. The veins, on their surface, injected with blood; on the side of each vein a track of reddened blood, about one-tenth of an inch wide.

The wind-pipe, as before remarked, was but slightly affected with inflammation, bnt about six inches from its ramifications into the lungs, it became extreme, the appearance being livid and blackish, but no raised or incipient sphacelating points, no coat of coagulating lymph. Two patches of mucus, similar to that which dropped from his mouth, or was coughed up, whilst living, found on its surface.

The kidneys were highly injected, but not inflamed, except that what usually resembles a pale coloured mucus was changed to a straw colour.

The mucous membrane of the bladder inflamed, rather than injected; the natural mucus increased in quantity; its outer surface not affected. The membrane lining the urethra, several inches from the bladder, in a high state of inflammation, but short of gangrene, rather high coloured than livid, not extending to the subjacent muscular coat.

The lungs natural, but high coloured.

The heart sound, injected, but flabby. The blood disposed to coagulate in the cavities; in one, as before remarked, a firm coagulum, the only one found.

The mucous membrane of the nose was not sufficiently affected to excite notice.

The colon and intestinal canal were unaffected on their inner surfaces.

One of the parts on the upper lip, which had been bitten, cut out, and had healed, as before stated, was now cut through in two directions; it exhibited no thickening to the eye, no hardness to the touch, and had the completest completest appearance of having healed by the first intention. During the disease, and to the last moment of his life, he had rubbed it against one thing or other with persevering eagerness, particularly during the exacerbations. This symptom had induced his owner, Mr. Townsend, to examine on Saturday evening the inside of his mouth, to discover whether there were any sore or hardness. He found none.

No inflammation of substance, or of the phlegmonous kind, was discovered; every diseased appearance was found in mucous membranes; and of those, except in the intestinal canal and nose, none of the larger ones had escaped; in no case, however, had the inflammation reached or attacked the subjacent coat.

The inflammation was most violent in the stomach, next in the wind-pipe, next in the urethra, next in the bladder. The strictured breathing might be imitated by compressing the throat externally by the hand; the violent inflammation, however, was many inches from the upper end of the neck.

During this examination, we were informed of a horse belonging to Mr. Teanby, builder, Old Street, supposed to be rabid from having been bitten by a dog. On inquiry, it was ascertained that he was bitten in Wormwood Street, probably by the same dog. He had completed twenty-two days, and part of twenty-three, since that time, before he had been much noticed; but the last mentioned day his work was suspended. It was now recollected that his appetite had failed since the twenty-first day. He was seen by the gentlemen above-mentioned about three in the afternoon of the twenty-fifth day.

On viewing him, little was found in his external appearance to notice, except a slight apparent uneasiness; a considerable degree of watchfulness as to surrounding objects, and apparently a disposition to stale frequently; whilst under examination, he voided to the amount of half a pint of urine at one time.

His usual temper was reported to be suspicious and churlish, so that the two former symptoms might be less unnatural to him than would be the case in a gentle horse. He bore the approach of his keeper quietly; suffered water to be splashed into his face without any apparent sensation beyond an ordinary start, and heard it poured from pail to pail without emotion; made attempts to drink without swallowing, or seeming to dread the water, and made many futile attempts to eat his hay; his desisting from eating, appeared rather the result of indifference, than of disgust.

The symptoms, compared with those of Mr. Townsend's horse, were so mild, that the writer was in no haste to see him in the early part of the following day. On calling about three in the afternoon he learnt, that in the morning he had become furious and ungovernable, biting the halter and the manger with vehemence, so that his owner had caused him to be shot for fear of farther mischief. He was already sent out of town to be buried, so that there was no opportunity of inspecting the carcase.

It has been already mentioned, that a horse belonging to Mr. Ashenden, was bit on the stand by Bishopsgate church. In about three-quarters of an hour, the bitten part was cut out by Mr. Waugh, farrier, Green Dragon Yard, Hog-lane, Curtain Road, from whom the following account was obtained: The bite was in the inside of the upper lip; it healed sound, without hardness or callus. The horse was low in condition, but whether of a gentle disposition is not ascertained; the symptoms were all mild; he was disposed to lie much; was rather stupid and quiet, and never snatched at any thing. He began to be ill on Tuesday, the 24th day from being bitten; he was then sent from work, weakly, and was recollected to have been off his appetite from Sunday the 22d day. From Wednesday the 24th, his desire to rub his lip was incessant; from the same time or earlier, showed a wish to drink, but never swallowed; breathed moderately; his flanks pinched to his back bone; no frothing at the mouth; no cough; drenched in perspiration from the beginning. He was killed on Saturday morning the 28th day, by repeated strokes on the head with a hammer.

Through the kindness of Mr. Jones, the writer proceeded to see him opened; but from some mistake, the parts were disposed of before he arrived. The wind-pipe and head alone were found, the former of a perfectly natural appearance throughout. The head exhibited marks of injury on the under jaws, as if he had bruised himself, but it is not ascertained that he had done so; the extravasated blood disposed to coagulate, and of a florid red.

The mare belonging to Mr. Crow was sent to the same farrier, Mr. Waugh, on account of an accidental lameness; she was with him when the symptoms first made their appearance. From him, up to Thursday evening, the twenty-sixth day from being bitten, the following account of the the symptoms was received. From that time to Saturday morning, the twenty-eighth, from Mr. Crow.

On finding her bitten, Mr. Crow instantly washed the place, one of the nostrils; it bled freely. The mare was, within an hour, dispatched to Mr. Waugh, who finding only a scratch, forthwith applied vitriolic acid as a caustic. On the morning of the twenty-sixth day he observed her unwell, but not greatly so. By request of her owner he gave her the Ormskirk medicine, which she had not had three hours before she began to paw and be uneasy.— She now began to breathe high; had no frothing at the mouth; staled freely previous to that day; afterwards, nothing farther could be learned in that respect; pinched in the flanks; perspired freely; and these symptoms continued to the last.

The eyes glassy, fiery, and red; the vessels considerably injected; began to bite at any thing in her way.

Though she had manifested a disposition so furious, yet she suffered herself to be led quietly to her master's stable on Thursday evening. The ensuing morning she became entirely ungovernable, and continued so as long as she was seen alive; indeed, in such a degree as to prevent all practibility of approach for accurate examination.

She was seen on Friday by Mr. Newington, surgeon, near Spital Square, through a fan-light over the door, and through which he introduced his hand, when she made a spring towards it, but failed, as she commonly did in such attempts, from apparent weakness of the back; shot forth her tongue and champed her teeth incessantly. She sprang forward with fury as often as any one came in sight; but being now, by the efforts of Mr. Crow's assistants, confined in a corner, they were able to offer her water as late as Friday morning and afternoon; she drank a gallon each time. In the afternoon, after drinking, she pawed over the rest with her foot. Eat nearly a peck of oats on Thursday night, and also some hay; a quantity of both were found in the stomach.

It should have been mentioned, that besides being confined in a corner by a gate, she had also a beast rope put round her neck, but without a slip noose. She was known to be alive on Saturday, the twenty-eighth morning, at half past six or seven o'clock: but she made so much noise, that those present were deterred from entering the stable till it was light. At eight she was found dead; but it could not be ascertained whether she might or might not have strangled herself. A slight extension of the inflammation from the internal to the external surface, as hereafter mentioned, tioned, of the trachea, might arise from this rope: but that can only be a conjecture.

The carcase was taken to an intelligent slaughterman in Whitechapel the same evening, where, for the alleged reason of preventing or retarding putrefaction, an incision had been made into the abdomen, and its contents drawn through it, were exposed to the air. The weather was frosty, which had apparently produced some effect on the viscera.

The examination was conducted under the inspection of Dr. Adams, Mr. Pettigrew, two other medical gentlemen, and the writer. The stomach was first subjected to inspection, and in it the most obvious appearance was, its inner surface adhering, from the frost or some other cause, to the masticated food, of which a large quantity was found. On turning this out, it stripped off about one half of its inner coat, leaving on the remainder, bounded by a jagged line of demarcation. The stripped surface looked red, but whether the muscular coat was exposed was not ascertained. The anatomy of the horse is not sufficiently known to the writer to enable him to say whether there be a cause for such a jagged line, and partial stripping of its coat, in the natural texture of the stomach; but he recollects an appearance precisely similar in the stomach of a horse which had died suddenly in pasture during the latter end of the year, and which was supposed to have died in consequence thereof; though the examination was not sufficiently accurate or minute to decide whether any other cause were discoverable.

The small intestines were affected in one place, but in no extensive degree, with inflammation of substance, including the peritonæal coat; this was the only inflammation of a serous membrane found, except such portions of the pleura as covered the inflamed bronchial tubes.

No other serous membranes, (and all were exposed to view) exhibited any preternatural appearance. The bladder was inflamed on its inner coat, as near as the writer could judge, in precisely the same way and degree as in Mr. Townsend's horse. The mucous membranes of the urethra inflamed through its length. In the bladder was found a small quantity of a jelly-like fluid. The uterus natural, perhaps flabby in its texture; the vagina natural; the brain healthy, as to its membranes and substance.

The chief seat of the disease was found to be the mucous membrane of the nose, trachea, and bronchia. The inflammation extended from the nasal surface of the cribriforme bone, completely enveloping the septum and turbinate binate bones, along the trachea, into every considerable bronchial ramification. The general colour of the inflammation on the interior surfaces or mucous membranes, was green, rather than livid, but partaking of both; and even such portions of the lungs and pleura as had taken it on, along with the subjacent and included bronchial tubes, were of the same colour; the lungs which had not been seized, were of a remarkably healthy appearance. The green livid inflammation of the substance of the lungs, had probably become injected in degree with coagulable lymph, being elastic, and rather firm on pressure; whereas, the healthy lungs were soft, yielding, and spongy as usual.

The injected vessels on the septum narium exhibited an interesting appearance, their larger ramifications being visible and numerous.

In one place, about midway down the trachea, the inflammation had attacked its cartilaginous and external surface; it did not appear to creep through the substance of the cartilage, but to have found its way by the connecting cellular membrane. Whether this might be caused by the halter, is not known; it might run from without, inwards.

The œsophagus was uninflamed, its lining of a pale and dingy colour, which we were informed was natural; the kidneys high coloured. The ureters not examined in this or the other horse.

No one being present to give information as to the situation of the bite, no particular notice was taken of the bitten part, it not being obvious on slight examination of the nose, where it was afterwards known to be. Mr. Jones, however, who saw the parts afterwards, says, the bitten part was thickened. It should have been mentioned, that in Mr. Ashenden's horse, the bitten part was thickened; his disposition to rub his nose incessant.

The last subject to be noticed was the horse belonging to Mr. Gray, the Royston carrier. Respecting him, only the following particulars could be ascertained. On his return homeward, the day on which he was bitten, he took the Puckeridge medicine, being met on the road with it at Hoddesdon.

He made his regular journeys to town, till Saturday, the 28th day, when he was found to be unwell. He, however, proceeded on his way to Hoddesdon, where it was judged prudent to leave him. He was said (for none of those of whom inquiries were made saw him) to be languid, dull, and affected with stupor and pinching-in of the flanks. He drank nearly a pail of water about three or four hours before before he died, and was said not to have staled during the complaint. He was suffered to die, which happened on Monday, the thirtieth from the bite, about eleven in the forenoon.

The only striking similarity in these cases, it would seem, is the seat of the disease, viz. the mucous membranes. The individual membranes affected, were not in both the same. In the horse, that of the stomach had suffered inflammation even to gangrene; in the mare no such appearance was found. The former neither eat nor drank, the latter did both; the œsophagus of the former slightly inflamed, of the latter not at all.

The appearances in the mucous membranes of the bladder of both were precisely similar. The necessary difference of structure in their respective urethras, prevented fair comparison; but as an opinion, the inflammation was the most considerable in that of the horse. He was known to suffer violent irritation in micturition; of the mare, no good account could be obtained in that respect.

In the mare, the whole of the mucous membrane from the cribriforme bone to the ends of the larger bronchial ramifications was highly inflamed; in the horse only five or six inches at the bronchial end, and that scarcely entering the ramifications.

The trachea shewn as that of Mr. Ashenden's horse, was perfectly sound its whole length; he was quiet during his complaint; Mr. Townsend's horse was nearly ungovernable, but the mare was furious. Will the comparative violence of the inflammation in their respective tracheas account for the difference?

No serous membrane was affected with inflammation, except where it had passed through; it did not appear, as to itself, to be a seat of the disease.

The incessant rubbing of the bitten parts would appear to demonstrate some connection of the uneasiness there, with the other symptoms; perspiration was so profuse in two of the horses and the mare, as to render it impossible to be overlooked. The brains of both healthy; the liver and spleen of both sound.

It may not be amiss to add a few other remarks to some of the writers of the present day. The coincidence of circumstances is sufficient to prove that the horses were all bitten by the same dog; and the variety of symptoms, as well as of the seat of the disease, in an animal that could not be governed by the imagination, may teach us to account for some varieties which have appeared in the human man subject; they at least place the question of the existence of such a disease beyond the sphere of fair scepticism. Inflammation of the mucous membranes is all that has been discovered. This affords but a glimmering towards accounting for the high nervous irritation which always precedes, and seems connected with, the cause of death.

Lastly, the number of subjects, the large scale of the various organs, the opportunity of comparing those which were diseased with similar parts in other animals of the same species, the medium period at which the symptoms appeared in each, and the attention with which every examination was conducted, will, it is hoped, justify the Writer in expressing a wish, that in every future examination, the membranes, mentioned in the account, will not be overlooked, whatever may have been the symptoms during life.

